# A215 DEFINING THE MODERN GLOBAL INCIDENCE AND PREVALENCE OF EOSINOPHILIC ESOPHAGITIS IN POPULATION-BASED STUDIES

**DOI:** 10.1093/jcag/gwad061.215

**Published:** 2024-02-14

**Authors:** C M Ray, K Buhler, C Ma

**Affiliations:** University of Calgary Cumming School of Medicine, Calgary, AB, Canada; University of Calgary Cumming School of Medicine, Calgary, AB, Canada; University of Calgary Cumming School of Medicine, Calgary, AB, Canada

## Abstract

**Background:**

Eosinophilic esophagitis (EoE) is an immune-mediated condition that results in esophageal dysfunction. Previous research demonstrated a marked increase in both incidence and prevalence of EoE globally, associated with a concomitant increase in type 2 inflammatory diseases. A previous meta-analysis suggested that the pooled prevalence of EoE was 34.4 cases/100,000 population and the incidence was 6.6 cases/100,000 person-years, but this analysis included only studies conducted to 2018.

**Aims:**

To assess the current global incidence and prevalence of EoE, through systematic review and meta-analysis of population-based studies.

**Methods:**

Electronic databases (Medline and Embase) were searched to February 22, 2023. Abstracts were screened, and full texts were reviewed for inclusion in meta-analysis. Original population-based studies published after 2018 that reported the incidence or prevalence of EoE in adults or children were included. Meta-analysis of incidence and prevalence rates, reported per 100,000 person-years or per 100,000 population, respectively, were pooled using a random-effects model. Heterogeneity was quantified using the *I*^*2*^ statistic.

**Results:**

A total of 1916 unique reports were identified, and abstracts were reviewed. 129 full texts were read, and 21 reports met inclusion criteria for population based, incidence and prevalence studies. The pooled global incidence of EoE was 8.43 cases per 100,000 person-years [95% CI: 6.52, 10.34] (I^2^=99%) (Figure 1). The incidence was similar in adult and pediatric subgroups. The pooled global prevalence of EoE was 68.0 cases per 100,000 population [95% CI: 37.5, 123.4] (I^2^=99%).

**Conclusions:**

The incidence and prevalence of EoE in recent studies is higher than that previously published. The prevalence of EoE is anticipated to compound, given that this is a disease typically diagnosed in young patients without an associated increased risk of mortality.

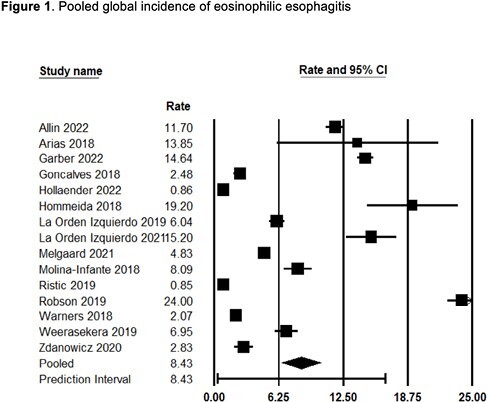

**Funding Agencies:**

None

